# A randomized, double‐blind, phase II study of oral histone deacetylase inhibitor resminostat plus S‐1 versus placebo plus S‐1 in biliary tract cancers previously treated with gemcitabine plus platinum‐based chemotherapy

**DOI:** 10.1002/cam4.3813

**Published:** 2021-02-26

**Authors:** Makoto Ueno, Chigusa Morizane, Masayuki Furukawa, Daisuke Sakai, Yoshito Komatsu, Yousuke Nakai, Masahiro Tsuda, Masato Ozaka, Nobumasa Mizuno, Manabu Muto, Akira Fukutomi, Masafumi Ikeda, Akihito Tsuji, Akio Katanuma, Toshikazu Moriwaki, Takeshi Kajiwara, Hiroshi Ishii, Yuji Negoro, Satoshi Shimizu, Noriko Nemoto, Shingo Kobayashi, Keigo Makino, Junji Furuse

**Affiliations:** ^1^ Department of Gastroenterology, Hepatobiliary and Pancreatic Medical Oncology Division Kanagawa Cancer Center Kanagawa Japan; ^2^ Department of Hepatobiliary and Pancreatic Oncology National Cancer Center Hospital Tokyo Japan; ^3^ Department of Hepato‐Biliary‐Pancreatology National Hospital Organization Kyushu Cancer Center Fukuoka Japan; ^4^ Department of Frontier Science for Cancer and Chemotherapy Osaka University Graduate School of Medicine Osaka Japan; ^5^ Division of Cancer Center Hokkaido University Hospital Hokkaido Japan; ^6^ Department of Gastroenterology Department of Endoscopy and Endoscopic Surgery Graduate School of Medicine The University of Tokyo Tokyo Japan; ^7^ Department of Gastroenterological Oncology Hyogo Cancer Center Hyogo Japan; ^8^ Hepato‐Biliary‐Pancreatic Medicine Department Cancer Institute Hospital of the Japanese Foundation for Cancer Research Tokyo Japan; ^9^ Department of Gastroenterology Aichi Cancer Center Hospital Aichi Japan; ^10^ Department of Clinical Oncology Kyoto University Hospital Kyoto Japan; ^11^ Division of Gastrointestinal Oncology Shizuoka Cancer Center Shizuoka Japan; ^12^ Department of Hepatobiliary and Pancreatic Oncology National Cancer Center Hospital East Chiba Japan; ^13^ Department of Clinical Oncology Faculty of medicine Kagawa University Kagawa Japan; ^14^ Center for Gastroenterology Teine‐keijinkai hospital Hokkaido Japan; ^15^ Department of Gastroenterology Faculty of Medicine University of Tsukuba Ibaraki Japan; ^16^ Department of Gastrointestinal Medical Oncology National Hospital Organization Shikoku Cancer Center Ehime Japan; ^17^ Clinical Research Center Chiba Cancer Center Chiba Japan; ^18^ Division of Clinical Oncology Kochi Health Sciences Center Kochi Japan; ^19^ Department of Gastroenterology Saitama Cancer Center Saitama Japan; ^20^ Pharmaceutical Research & Development Department Yakult Honsha Co., Ltd. Tokyo Japan; ^21^ Department of Medical Oncology Faculty of Medicine Kyorin University Tokyo Japan

**Keywords:** biliary tract cancers, histone deacetylase inhibitor, resminostat plus S‐1, systemic chemotherapy

## Abstract

**Purpose:**

Effective second‐line chemotherapy options are limited in treating advanced biliary tract cancers (BTCs). Resminostat is an oral histone deacetylase inhibitor. Such inhibitors increase sensitivity to fluorouracil, the active form of S‐1. In the phase I study, addition of resminostat to S‐1 was suggested to have promising efficacy for pre‐treated BTCs. This study investigated the efficacy and safety of resminostat plus S‐1 in second‐line therapy for BTCs.

**Methods:**

Patients were randomly assigned to receive resminostat or placebo (200 mg orally per day; days 1–5 and 8–12) and S‐1 group (80–120 mg orally per day by body surface area; days 1–14) over a 21‐day cycle. The primary endpoint was progression‐free survival (PFS). Secondary endpoints comprised overall survival (OS), response rate (RR), disease control rate (DCR), and safety.

**Results:**

Among 101 patients enrolled, 50 received resminostat+S‐1 and 51 received placebo+S‐1. Median PFS was 2.9 months for resminostat+S‐1 vs. 3.0 months for placebo+S‐1 (HR: 1.154, 95% CI: 0.759–1.757, *p* = 0.502); median OS was 7.8 months vs. 7.5 months, respectively (HR: 1.049, 95% CI: 0.653–1.684, *p* = 0.834); the RR and DCR were 6.0% vs. 9.8% and 70.0% vs. 78.4%, respectively. Treatment‐related adverse events (TrAEs) of grade ≥ 3 occurring more frequently (≥10% difference) in the resminostat+S‐1 than in the placebo+S‐1 comprised platelet count decreased (18.0% vs. 2.0%) and decreased appetite (16.0% vs. 2.0%).

**Conclusions:**

Resminostat plus S‐1 therapy improved neither PFS nor OS for patients with pre‐treated BTCs. Addition of resminostat to S‐1 was associated with higher incidence of TrAEs, but these were manageable (JapicCTI‐183883).

## Graphical abstract

This randomized, placebo‐controlled, double‐blind study investigated the efficacy and safety of resminostat plus S‐1 as a second‐line therapy in patients with BTCs. This was the first randomized study to use S‐1, which is widely used in second‐line treatment for BTC patients in Japan, as an active comparator. The results showed that resminostat plus S‐1 prolonged neither PFS nor OS in comparison with placebo plus S‐1.

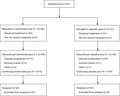

## 1 INTRODUCTION

Although biliary tract cancers (BTCs) occur infrequently, the mortality rate is high.^1^ In Japan, gallbladder and bile duct cancers were ranked sixth as the cause of cancer death in 2018.^2^ The only radical treatment available for BTCs is surgical intervention. Because BTCs are often already unresectable by the time they are diagnosed, this option is not feasible in such patients. Even after curative surgery, the rate of recurrence is high, conferring a poor prognosis.^3^ Therefore, systemic chemotherapy is considered to be the first therapeutic option in treating patients with advanced BTCs.

The standard first‐line chemotherapy regimen for locally advanced or metastatic BTCs is gemcitabine plus cisplatin (GC).^4^ In recent years, two Japanese phase III studies (JCOG1113 and KHBO1401‐MITSUBA) have shown the benefits of gemcitabine or GC in combination with the oral fluoropyrimidine S‐1 as first‐line regimens for treating advanced/recurrent BTCs.^5^, ^6^ These two regimens have become new therapeutic options for previously untreated BTCs in Japan. Currently, there is no global standard of care for second‐line treatment in BTC patients. S‐1 monotherapy has widely been used in this setting in Japan, however, due to favorable results from single‐arm studies of second‐line S‐1 monotherapy.^7^, ^8^ A recent UK‐based phase III study (ABC‐06) demonstrated the benefits of combining fluorouracil with leucovorin and oxaliplatin (mFOLFOX6) over active symptom control in second‐line BTC treatment for progressive disease following GC therapy.^9^ Whether second‐line FOLFOX is more effective than fluoropyrimidine treatment alone (e.g., S‐1) in the patients with BTCs remains unclear.^10^ Hence, second‐line therapeutic options for pre‐treated BTCs are limited.

Resminostat inhibits class I, IIb, and IV histone deacetylases (HDACs), which function as epigenetic regulators. By acting on the histones in the nucleosome, they modulate the structure of chromatin, regulating the expression of a variety of genes involved in the control of cell survival, proliferation, differentiation, and apoptosis.^11^, ^12^ HDACs are overexpressed in a wide variety of cancers, including BTCs, and data have suggested that their overexpression is associated with more advanced disease and poorer prognosis.^13^, ^14^

Fluorouracil, the active form of S‐1, inhibits deoxyribonucleic acid biosynthesis by forming a ternary complex with thymidylate synthase (TS) and a reduced form of folic acid.^15^ TS controls the tumor cell sensitivity to fluorouracil. Preclinical data suggested that repeated exposure of tumor cells to fluorouracil enhances the expression of TS, increasing resistance to fluorouracil.^15^, ^16^ Another study also reported that patients expressing high levels of TS were resistant to S‐1 therapy.^17^ It has been suggested that HDAC inhibitors increase the sensitivity of lung cancer cell lines to fluorouracil by suppressing expression of TS.^18^ These findings have led to the hypothesis that the addition of resminostat to S‐1 would result in enhanced antitumor activity. Patients with BTCs receiving resminostat plus S‐1 as second or subsequent therapy in a previous phase I study showed a median progression‐free survival (PFS) of 5.5 months and median overall survival (OS) of 10.2 months.^19^ These outcomes were more favorable than those seen with various other second‐line treatments for BTC patients (median PFS of 3.2 months; median OS of 7.2 months) according to one meta‐analysis.^20^ The purpose of the present phase II study was to compare the efficacy and safety of resminostat plus S‐1 with those of placebo plus S‐1 in second‐line therapy for BTC patients with disease progression following treatment with a gemcitabine plus platinum‐based regimen.

## 2 MATERIALS AND METHODS

### 2.1 Patients

The eligibility criteria included the following: unresectable/recurrent BTCs, including cancers of the intra‐ or extrahepatic bile ducts, the gallbladder, and the ampulla of Vater; pathologically confirmed adenocarcinoma; only one prior systemic chemotherapy regimen consisting of gemcitabine and a platinum agent; disease progression confirmed by the investigator based on available imaging reports; at least one measurable tumor lesion according to the Response Evaluation Criteria in Solid Tumors (RECIST) version 1.1; Eastern Cooperative Oncology Group performance status (ECOG PS) of 0 or 1; age 20–79 years; life expectancy of at least 12 weeks; and adequate organ and bone marrow function (hemoglobin ≥9.0 g/dl, neutrophil count ≥1500/mm^3^, platelet count ≥100,000/mm^3^, aspartate transaminase and alanine transaminase ≤2.5 × the institutional upper limit of normal, serum total bilirubin ≤2.0 mg/dl, serum creatinine ≤1.5 mg/dl, creatinine clearance ≥60 ml/min, and Fridericia‐corrected QT interval <460 msec).

The exclusion criteria included the following: prior treatment with HDAC inhibitors, prior fluoropyrimidine treatment (except for adjuvant or neoadjuvant chemotherapy), prior radiation therapy for BTCs, history of myocardial infarction within 6 months prior to enrollment or cardiovascular complications, ascites requiring treatment, clinically significant bone metastasis, and known or suspected brain metastasis.

The protocol of this study received approval from the institutional review board of each participating site. It was performed according to the requirements of Good Clinical Practice and the Declaration of Helsinki. Before enrollment, all patients provided written informed consent.

### 2.2 Study design

This was a multi‐center, randomized, placebo‐controlled, double‐blind, phase II study (registered with JAPIC Clinical Trials Information, identifier: JapicCTI‐183883). It was performed at 21 sites in Japan. The patients were randomly assigned in a ratio of 1:1 to receive resminostat+S‐1 or placebo+S‐1 by the minimization method, with stratification according to site, primary tumor site (gallbladder vs. others), a history of postoperative recurrence (yes vs. no), and ECOG PS (0 vs. 1). Enrollment and assignment were performed using the interactive web response system.

The primary endpoint comprised PFS as assessed by the investigator; secondary endpoints were OS, response rate (RR), disease control rate (DCR), and safety.

### 2.3 Treatments

Each patient was scheduled to receive either resminostat 200 mg or placebo once daily after meals on days 1–5 and 8–12 over a 21‐day cycle. S‐1 (80 mg/day for body surface area [BSA] of <1.25 m^2^, 100 mg/day for BSA of 1.25 to <1.50 m^2^, and 120 mg/day for BSA of ≥1.50 m^2^) was administered twice daily after meals on days 1–14.

Treatment was to be continued unless disease progression, consent withdrawal, unacceptable toxicity, or criteria indicating the need for discontinuation were observed. Resminostat or placebo was discontinued if grade ≥3 QT interval prolongation developed. The doses of resminostat/placebo were reduced if grade 4 platelet count decreased and developed. The dose of S‐1 was reduced for grade 4 platelet count decreased/neutrophil count decreased or for grade ≥3 mucositis oral/diarrhea. If other grade ≥3 clinically significant adverse events (AEs) occurred, the dose of the drug judged as being the more likely cause of the AE by the investigator was reduced. No dose escalation of resminostat/placebo or S‐1 after reduction was permitted.

### 2.4 Assessment

Tumor response was evaluated by each investigator in accordance with RECIST version 1.1 at before enrollment, at weeks 6, 12, 18, and 24, and then every 8 weeks thereafter until progressive disease. Assessment of AEs was performed according to the Common Terminology Criteria for Adverse Events version 4.0.

### 2.5 Statistical analysis

PFS in placebo+S‐1 was expected to be 3.0 months based on previous studies of S‐1 as second‐line therapy for BTCs and a meta‐analysis of second‐line therapies for BTCs.^7^, ^20^ In resminostat+S‐1, PFS was expected to be 5.0 months based on the previous phase I study of the same agents.^19^ With a one‐sided significance level of 10% and a statistical power of 80%, 71 PFS events were required. Assuming a 12‐month accrual and a minimum follow‐up of 6 months from enrollment of the last patient, 82 patients were needed. Considering that some patients would be lost to follow‐up, 100 patients were planned to be included in this study.

The safety analysis targeted all patients receiving a minimum of one dose of any of the study drugs. The efficacy analyses were based on the full analysis set comprising all patients meeting the study eligibility criteria in the safety analysis population.

The following definitions were set: PFS, time from enrollment date to disease progression or death from any cause; OS, time from enrollment date to death from any cause. The log‐rank test was used to make a comparison of PFS and OS between treatment groups. Median PFS, median OS, and their two‐sided 95% confidence intervals (CIs) for each treatment group were estimated using the Kaplan–Meier method. The hazard ratios (HRs) and their 95% CIs were calculated using Cox regression analysis. Subgroup analyses were preplanned to explore the heterogeneity of PFS and OS in each subgroup according to patient characteristics and baseline tumor size. The RR (the proportion of patients whose best overall response was either complete response [CR] or partial response [PR]), the DCR (the proportion of patients whose best overall response was CR, PR, or stable disease), and each 95% CI was calculated for an inter‐group comparison.

The primary analysis was conducted at 6 months following the date on which the last patient was enrolled (data cut‐off: August 24, 2019). All statistical analyses were carried out using SAS version 9.3 or 9.4 (SAS Institute).

## 3 RESULTS

### 3.1 Patients

**FIGURE 1:**
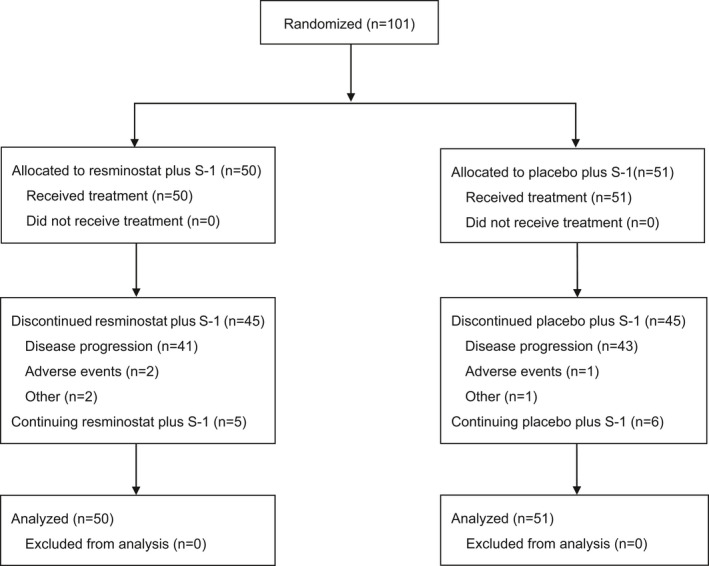
Patient flow diagram

Between March 2018 and February 2019, 101 patients were enrolled in this study, of which 50 were randomly assigned to resminostat+S‐1 and 51 to placebo+S‐1 (Figure 1). All 101 patients received the study treatments. Baseline characteristics between groups were observed to be well balanced (Table 1).

**TABLE 1:** Baseline characteristics

	Resminostat+S‐1 (*N* = 50)		Placebo+S‐1 (*N* = 51)	
	*N*	%	*N*	%
Sex				
Male	28	56.0	28	54.9
Female	22	44.0	23	45.1
Race				
Asian	50	100.0	51	100.0
Age (years)				
Median	64.5		67.0	
Range	32–79		39–78	
Performance status				
0	37	74.0	36	70.6
1	13	26.0	15	29.4
Primary tumor site				
Intrahepatic bile duct	23	46.0	19	37.3
Extrahepatic bile duct	8	16.0	17	33.3
Gallbladder	13	26.0	10	19.6
Ampulla of Vater	6	12.0	5	9.8
Histopathological diagnosis				
Adenocarcinoma	50	100.0	51	100.0
Disease status				
Recurrence	16	32.0	19	37.3
Locally advanced	5	10.0	5	9.8
Metastasis	29	58.0	27	52.9
Biliary drainage				
No	30	60.0	23	45.1
Yes	20	40.0	28	54.9
Prior chemotherapy				
Gemcitabine plus cisplatin^a^	50	100.0	51	100.0
S‐1^b^	1	2.0	1	2.0
Number of target lesions				
1	20	40.0	20	39.2
≥2	30	60.0	31	60.8

^a^ One patient in placebo+S‐1 was treated with gemcitabine+cisplatin as an adjuvant therapy.

^b^ Two patients treated with S‐1 as an adjuvant therapy subsequently received gemcitabine+cisplatin as first‐line therapy after recurrence.

### 3.2 Treatments

The median number of treatment cycles was 4 (range: 1–18) in resminostat+S‐1 and 4 (range: 1–21) in placebo+S‐1. The median relative dose intensity of resminostat/placebo was 82.4% (range: 25.0–100.0) in resminostat+S‐1 and 85.2% (range: 28.0–100.0) in placebo+S‐1, while the median relative dose intensity of S‐1 was 80.2% (range: 17.8–100.0) and 80.0% (range: 25.8–100.0), respectively. Eleven patients (10.9%) were still receiving the study treatment (five patients [10.0%] in resminostat+S‐1 and six patients [11.8%] in placebo+S‐1) at the data cut‐off. Disease progression was the most frequent reason for treatment discontinuation in both groups (41 of 45 patients [91.1%] in resminostat+S‐1 and 43 of 45 patients [95.6%] in placebo+S‐1).

### 3.3 Efficacy

**FIGURE 2:**
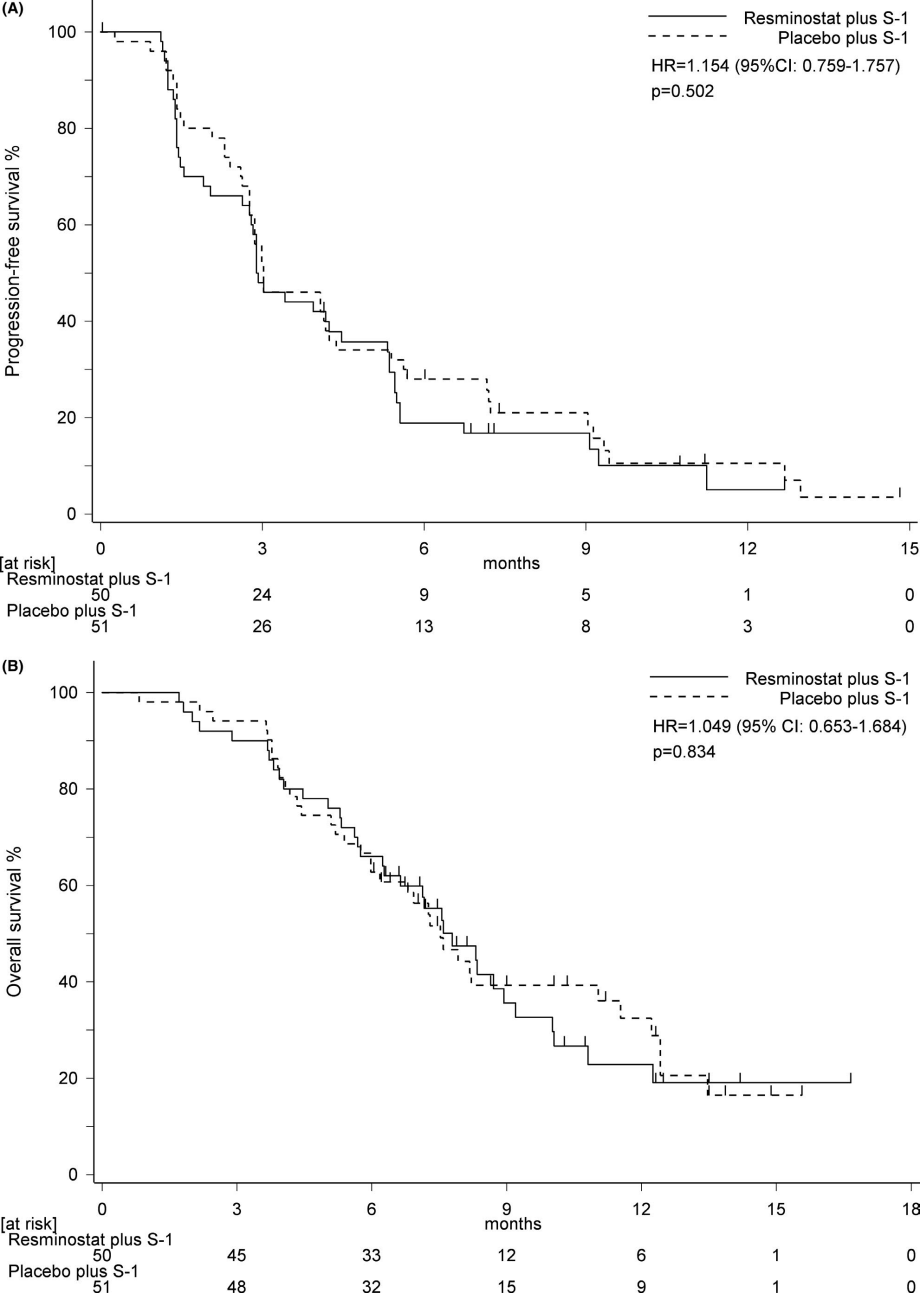
Progression‐free survival and overall survival (A) Progression‐free survival and (B) overall survival in full analysis set by Kaplan–Meier method. HR, hazard ratio; CI, confidence interval

Median follow‐up for PFS was 7.2 months (95% CI: 4.1–12.7) in resminostat+S‐1 and 6.7 months (95% CI: 0.0–14.8) in placebo+S‐1. Median PFS was 2.9 months (95% CI: 2.6–4.5) in resminostat+S‐1 compared with 3.0 months (95% CI: 2.8–4.2) in placebo+S‐1 (HR: 1.154, 95% CI: 0.759–1.757, *p* = 0.502) (Figure 2A).

Median follow‐up for OS was 8.0 months (95% CI: 7.1–12.3) in resminostat+S‐1 and 9.5 months (95% CI: 6.8–12.3) in placebo+S‐1. Median OS was 7.8 months (95% CI: 6.2–9.2) in resminostat+S‐1 compared with 7.5 months (95% CI: 6.0–11.5) in placebo+S‐1 (HR: 1.049, 95% CI: 0.653–1.684, *p* = 0.834) (Figure 2B).

Three patients (6.0%, 95% CI: 1.3–16.5) in resminostat+S‐1 and five patients (9.8%, 95% CI: 3.3–21.4) in placebo+S‐1 achieved PR (*p* = 0.715), with no patient in either group achieving CR.

**FIGURE 3:**
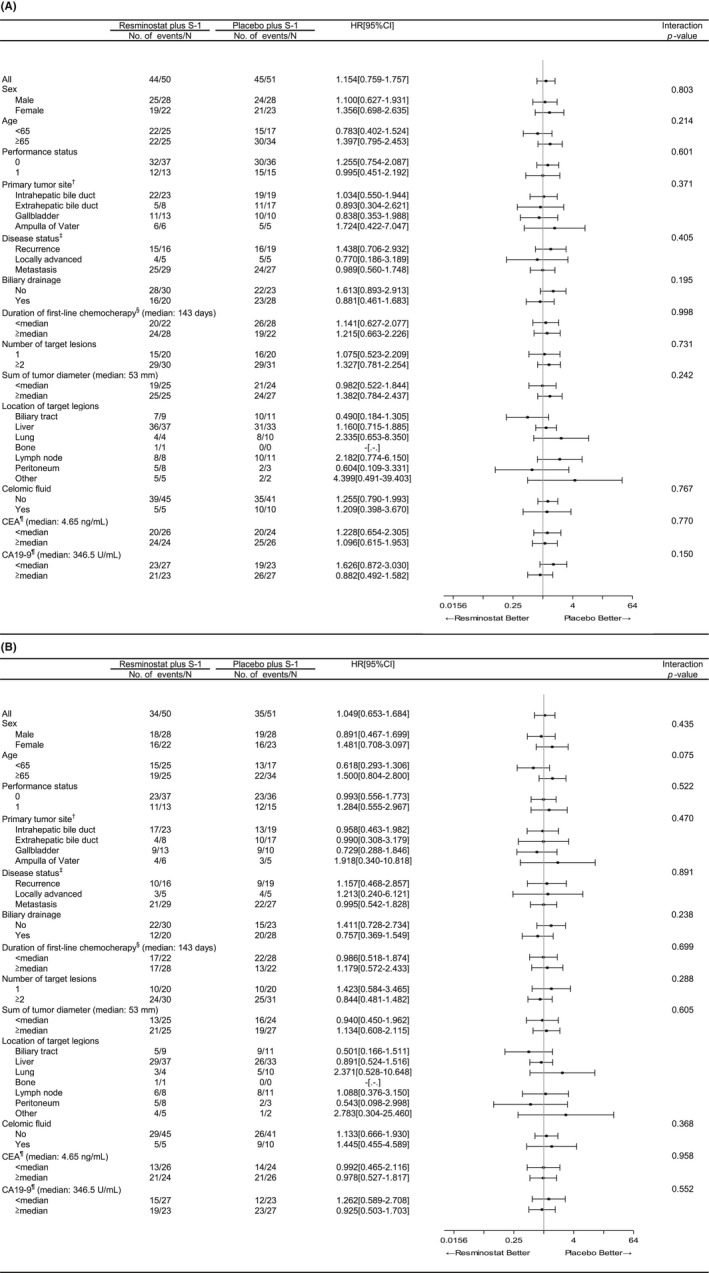
(A) Subgroup analysis of progression‐free survival. (B) Subgroup analysis of overall survival. HR, hazard ratio; CI, confidence interval. ^†^Primary tumor site was categorized as gallbladder or others in calculating interaction *p*‐value. ^‡^Disease status was categorized as recurrence or others in calculating interaction *p*‐value. ^§^One patient in placebo+S‐1 treated with gemcitabine+cisplatin as an adjuvant therapy was excluded from calculation. ^¶^Data were missing for one patient in placebo+S‐1

DCR was 70.0% (95% CI: 55.4–82.1) in resminostat+S‐1 and 78.4% (95% CI: 64.7–88.7) in placebo+S‐1 (*p* = 0.369) (Table 2). In pre‐specified subgroup analyses for PFS and OS, no survival benefit was observed in any subgroup (Figure 3).

**TABLE 2:** Efficacy outcomes

		Resminostat+S‐1		Placebo+S‐1	
		(*N* = 50)		(*N* = 51)	
		*N*	%	*N*	%
Best overall response	Complete response	0	0.0	0	0.0
	Partial response	3	6.0	5	9.8
	Stable disease	32	64.0	35	68.6
	Progressive disease	15	30.0	10	19.6
	Not evaluable	0	0.0	1	2.0
Response rate	% [95% CI]	6.0 [1.3–16.5]		9.8 [3.3–21.4]	
	*p*‐value	0.715			
Disease control rate	% [95% CI]	70.0 [55.4–82.1]		78.4 [64.7–88.7]	
	*p*‐value	0.369			
Time to response^a^ (months)	Median [95% CI]	1.5 [1.4–2.9]		2.8 [1.2–14.8]	
Duration of response^a^ (months)	Median [95% CI]	NR [2.6‐NR]		7.3 [4.4–8.2]	

Abbreviations: CI, confidence interval; NR, not reached.

^a^ Time to response and duration of response were evaluated in the patients who were achieved partial response or better.

### 3.4 Safety

Treatment‐related AEs (TrAEs) with an incidence of ≥10% in either group are shown in Table 3. The TrAEs of any grade that occurred more frequently (≥10% difference) with resminostat+S‐1 than placebo+S‐1 comprised platelet count decreased (76.0% vs. 49.0%), nausea (72.0% vs. 41.2%), decreased appetite (64.0% vs. 41.2%), vomiting (46.0% vs. 13.7%), and dysgeusia (32.0% vs. 21.6%), respectively. The incidence of grade ≥3 TrAEs was higher in resminostat+S‐1 than in placebo+S‐1 (54.0% vs. 29.4%). The grade ≥3 TrAEs that occurred more frequently (≥10% difference) with resminostat+S‐1 vs. placebo+S‐1 were platelet count decreased (18.0% vs. 2.0%) and decreased appetite (16.0% vs. 2.0%). The incidence of serious TrAEs was similar between the treatment groups (14.0% in resminostat+S‐1 vs. 13.7% in placebo+S‐1).

**TABLE 3:** Treatment‐related adverse events (TrAEs) reported in ≥10% of patients in either group

TrAEs	Resminostat+S‐1 (*N* = 50)				Placebo+S‐1 (*N*= 51)			
	All grade		≥Grade 3		All grade		≥Grade 3	
	*N*	%	*N*	%	*N*	%	*N*	%
Any	49	98.0	27	54.0	48	94.1	15	29.4
Platelet count decreased	38	76.0	9	18.0	25	49.0	1	2.0
Nausea	36	72.0	2	4.0	21	41.2	1	2.0
Decreased appetite	32	64.0	8	16.0	21	41.2	1	2.0
Vomiting	23	46.0	0	0.0	7	13.7	0	0.0
Neutrophil count decreased	19	38.0	10	20.0	16	31.4	6	11.8
Dysgeusia	16	32.0	0	0.0	11	21.6	0	0.0
Anemia	15	30.0	5	10.0	17	33.3	6	11.8
White blood cell count decreased	14	28.0	5	10.0	12	23.5	2	3.9
Stomatitis	13	26.0	1	2.0	13	25.5	0	0.0
Malaise	12	24.0	0	0.0	13	25.5	0	0.0
Diarrhea	11	22.0	2	4.0	11	21.6	1	2.0
Fatigue	9	18.0	2	4.0	9	17.6	1	2.0
Lymphocyte count decreased	9	18.0	4	8.0	5	9.8	2	3.9
Skin hyperpigmentation	8	16.0	0	0.0	10	19.6	0	0.0
Pyrexia	6	12.0	0	0.0	2	3.9	0	0.0
Weight decreased	6	12.0	1	2.0	2	3.9	0	0.0
Blood creatinine increased	5	10.0	0	0.0	2	3.9	0	0.0
Rash	3	6.0	0	0.0	7	13.7	0	0.0
Lacrimation increased	1	2.0	0	0.0	7	13.7	0	0.0

No cardiac TrAEs occurred in resminostat+S‐1. Grade 2 electrocardiogram QT prolonged was reported in one patient in placebo+S‐1; this was the only cardiac TrAE reported in this study.

Treatment was discontinued due to a TrAE in a total of three patients. The TrAEs that required discontinuation of the study treatment were tumor hemorrhage (one patient [2.0%]) and pneumonitis (one patient [2.0%]) in resminostat+S‐1, and leukoencephalopathy (one patient [2.0%]) in placebo+S‐1.

Treatment‐related death occurred in one patient in resminostat+S‐1 due to tumor hemorrhage.

## 4 DISCUSSION

The aim of this randomized study was to determine whether adding the HDAC inhibitor resminostat to oral fluoropyrimidine S‐1 improved survival outcome in BTC patients after failure of gemcitabine plus platinum‐based first‐line therapy. The results demonstrated that resminostat+S‐1 prolonged neither PFS nor OS in comparison with placebo+S‐1 in second‐line treatment for advanced or recurrent BTCs. Placebo+S‐1 showed a median PFS of 3.0 months, which is identical to that expected with S‐1 monotherapy. Resminostat+S‐1 showed a median PFS of only 2.9 months. The result did not support the findings from the phase I study^19^ and indicates that the addition of resminostat to S‐1 does not confer a clinical benefit in patients with BTCs compared to S‐1 monotherapy.

Various prognostic factors, such as ECOG PS, history of resection, and tumor markers, have been reported to be associated with survival in patients with BTCs.^21^, ^22^ Therefore, we reviewed if some of these factors affected the results of the present study. The results of Cox regression analyses showed that certain parameters, including some of those identified above in earlier studies, were prognostic factors (Table S1). However, no significant imbalance was observed between the two groups with respect to baseline characteristics in this study. This suggests that it is unlikely that any baseline characteristics affected the study results.

We expected that combining resminostat with S‐1 would result in a synergistic effect manifesting in suppression of expression of TS. However, tumor resistance to fluorouracil agents was reported to be due to not only up‐regulation of TS but also other mechanisms.^23^ Merely suppressing TS alone might be insufficient to overcome resistance.

In this study, the daily dose of 200 mg resminostat (days 1–5 and 8–12) in combination with S‐1 as the recommended regimen for phase II was selected based on the results of a previous study.^19^ On the other hand, maximum HDAC activity inhibition with resminostat had been obtained at doses of 400 mg/day or higher.^24^ The recommended dose of resminostat as a single agent was reported to be 800 mg/day in Japanese patients with solid tumors.^25^ Although the phase I study for patients with pre‐treated biliary tract or pancreatic cancer supported the lower dose, it might be insufficient to exert an add‐on effect to S‐1.

HDAC inhibitors have demonstrated clinical benefits in some types of hematological malignancy. Pan‐HDAC inhibitors, including vorinostat and panobinostat, were approved for use in cutaneous T‐cell lymphoma and multiple myeloma. This suggests that differences in molecular background such as the frequency of MYC gene abnormality between hematological malignancies and BTCs^26^, ^27^ might affect the mechanism of HDAC inhibitors. In the present study, no marked findings were obtained to evaluate the influence of molecular background. Further studies including biomarker analyses are warranted.

Gastrointestinal TrAEs (e.g., nausea, vomiting, and decreased appetite) as well as platelet count decreased were observed more frequently in resminostat+S‐1. These toxicities were similar in nature to those reported in the previous phase I study^19^ and could be managed well by the dose reductions/interruptions and/or antiemetic support. None of these common TrAEs led to discontinuation of resminostat+S‐1 therapy. Although cardiac toxicity has been reported to be associated with HDAC inhibitors,^12^ no cardiac TrAEs occurred in resminostat+S‐1, suggesting that the resminostat does not cause cardiac toxicities at the dose level used in this study. Just one patient in resminostat+S‐1 died from tumor hemorrhage. The cause of death in this case was deemed to be related to the study treatment by the investigator. Although it is unclear as to whether the tumor hemorrhage resulted from disease progression or some tumor response to treatment, the resminostat‐induced platelet count decreased observed in this patient may have prevented hemostasis, leading to the fatal outcome.

To the best of our knowledge, this was the first randomized study to use S‐1, a drug commonly used in second‐line treatment of BTCs in Japan, as an active comparator. We are sure that the outcomes observed in placebo+S‐1 will provide a valuable reference for future studies. Recently, the median OS in an active symptom control plus mFOLFOX6 group was reported to be 6.2 months in the ABC‐06 study.^9^ Although outcomes from the study cannot be compared directly with our data, our results suggested that patients with BTCs may receive a survival benefit from S‐1 monotherapy. However, effective therapeutic options are still limited in patients with second‐line BTCs.

In summary, the results of this study indicated that resminostat plus S‐1 therapy was not an effective second‐line treatment for unresectable or recurrent BTCs. The number of TrAEs, especially gastrointestinal toxicity and platelet count decreased, with resminostat plus S‐1 therapy was higher than those with placebo plus S‐1 therapy. However, the safety profile with this combination therapy was consistent with that in previous studies, and the TrAEs were manageable.

## CONFLICT OF INTERESTS

MU reports grants and personal fees from Yakult during the conduct of the study; grants and personal fees from Taiho, AstraZeneca, Merck Serono, MSD, Daiichi Sankyo, Ono, personal fees from Nihon Servier, grants from Astellas, Eisai, Sumitomo Dainippon, Incyte outside the submitted work. CM reports grants and personal fees from Taiho, Yakult, Nobelpharma, personal fees from Novartis, AbbVie, Fujifilm, Teijin, grants from Eisai, Pfizer, Ono outside the submitted work. DS reports grants and personal fees from Chugai, grants from Yakult, Ono, Eli Lilly, Daiichi Sankyo, Astellas, Incyte outside the submitted work. YK reports grants and personal fees from Yakult during the conduct of the study; grants and personal fees from Asahi Kasei, Bayer, Daiichi Sankyo, Ono, Taiho, personal fees from Bristol‐Myers Squibb, Chugai, Eli Lilly, Kyowa Kirin, Medical Review, Merck Biopharma, Mitsubishi Tanabe, Moroo, Nipro, Pfizer, Sanofi, Shire, grants from A2 Healthcare, Astellas, Sumitomo Dainippon, Eisai, Mediscience Planning, NanoCarrier, Parexel, Sanofi, Shionogi, Incyte, IQVIA, MSD, Nippon Zoki, Syneos Health Clinical, Sysmex outside the submitted work. YN reports grants and personal fees from Yakult, Taiho outside the submitted work. MO reports grants and personal fees from Yakult during the conduct of the study; grants and personal fees from Taiho, personal fees from Bayer, Pfizer, Novartis, Takeda, Eisai, EA pharma, Mitsubishi Tanabe, grants from Merck, Incyte, ASLAN outside the submitted work. NM reports grants from Yakult during the conduct of the study; grants and personal fees from Novartis, Taiho, AstraZeneca, MSD, personal fees from Yakult, Teijin, grants from Eisai, Sumitomo Dainippon, ASLAN, Incyte, Ono outside the submitted work. MM reports grants and other from Chugai, grants from Taiho, Sysmex, Riken Genesis, Mitsui Knowledge, other from Yakult outside the submitted work. AF reports grants from Yakult during the conduct of the study; grants and personal fees from Taiho, Teijin, personal fees from Nihon Servier, Shire, Yakult, grants from Sumitomo Dainippon, NanoCarrier, Aslan, Incyte Biosciences Japan outside the submitted work. MI reports grants and non‐financial support from Yakult during the conduct of the study; grants and personal fees from Bayer, Eisai, Eli Lilly Japan, Chugai, AstraZeneca, MSD, Takeda, Nano Carrier, ASLAN, personal fees from Sumitomo Dainippon, Taiho, Nihon Servier, Teijin, Mylan, Astellas, EA Pharma, Shire, Otsuka, grants from Novartis, Bristol‐Myers Squibb, Merck Serono, Ono, J‐Pharma, Pfizer outside the submitted work. AT reports grants from Yakult during the conduct of the study; grants and personal fees from Taiho, Chugai, Eli Lilly, Merck Biopharma, Takeda, Sanofi, personal fees from Bristol‐Myers Squibb, grants from Ono, Kyowa Kirin, Eisai, Toray Medical, Daiichi Sankyo, Bayer, Shionogi, Pfizer, Yakult outside the submitted work. TM reports grants and personal fees from Yakult during the conduct of the study; grants and personal fees from Takeda, Taiho, personal fees from Chugai, Eli Lilly, Bayer, Sanofi, Ono, grants from MSD, Eisai outside the submitted work. TK reports personal fees from Chugai, Taiho, Bristol‐Myers Squibb, Merck Biopharma, Kyowa Kirin outside the submitted work. HI reports personal fees from Yakult outside the submitted work. SS reports grants from Yakult during the conduct of the study. NN, SK and KM are employees of Yakult. NN and KM own stock in Yakult. JF reports grants and personal fees from Yakult during the conduct of the study; personal fees from Eisai, Bayer, Taiho, Ono, Novartis, Yakult, Teijin, Shionogi, EA pharma, Eli Lilly, Takeda, Chugai, Mochida, Nihon Servier, Sanofi, Fujifilm Toyama Chemical, Nobel pharma, Pfizer, Sawai, Daiichi Sankyo, Sumitomo Dainippon, Merck Serono, Nippon Kayaku, MSD, Shire, Kyowa Kirin, grants from Ono, MSD, Sumitomo Dainippon, J‐Pharma, Yakult, AstraZeneca, Daiichi Sankyo, Eisai, Bayer, Pfizer, NanoCarrier, Kyowa Kirin, Taiho, Chugai, Sanofi, Takeda, Mochida, Astellas, Eli Lilly outside the submitted work.

## Supporting information

Table S1

## Data Availability

Research data are not shared.
