# Supplementary material for: A randomized, double‐blind, phase II study of oral histone deacetylase inhibitor resminostat plus S‐1 versus placebo plus S‐1 in biliary tract cancers previously treated with gemcitabine plus platinum‐based chemotherapy
Source: Cancer Med. 2021 Feb 26;10(6):2088–99. doi: 10.1002/cam4.3813 (PMC7957161; doi:10.1002/cam4.3813)
Supplement: Supplementary file 1 — Table S1 [file CAM4-10-2088-s001.pdf]

Table S1 Multivariate Cox regression analysis for progression-free survival and overall survival

	Variables†	Category	HR	95%CI	*p*-value
PFS	Group	resminostat+S-1 (versus placebo+S-1)	1.376	0.885-2.140	0.156
	Age	≥65 (versus <65)	1.483	0.882-2.493	0.136
	Eastern Cooperative Oncology Group performance status	1 (versus 0)	1.790	1.060-3.024	0.029
	Primary tumor site	Gallbladder (versus Others)	1.514	0.876-2.616	0.136
	Number of target lesions	≥2 (versus 1)	2.725	1.648-4.506	<0.001
	Celomic fluid	Yes (versus No)	2.734	1.464-5.105	0.001
	CEA (median: 4.65 ng/mL)	≥median (versus <median)	1.639	0.978-2.747	0.060
	CA19-9 (median: 346.5 U/mL)	≥median (versus <median)	2.275	1.398-3.702	<0.001
OS	Group	resminostat+S-1 (versus placebo+S-1)	1.265	0.749-2.139	0.379
	Age	≥65 (versus <65)	1.981	1.093-3.590	0.024
	Disease status (Recurrence)	Yes (versus No)	0.624	0.348-1.120	0.114
	Duration of first-line chemotherapy (median: 143 days)	≥median (versus <median)	0.500	0.293-0.854	0.011
	Number of target lesions	≥2 (versus 1)	3.912	2.124-7.205	<0.001
	Celomic fluid	Yes (versus No)	3.316	1.708-6.439	<0.001
	CEA (median: 4.65 ng/mL)	≥median (versus <median)	2.145	1.183-3.889	0.011
	CA19-9 (median: 346.5 U/mL)	≥median (versus <median)	2.535	1.416-4.539	0.001

PFS, Progression-free survival; OS, overall survival; HR, hazard ratio; CI, confidence interval.

† Variables selected by backward elimination method (level of significance greater than 0.2 if explanatory variables excluded).
The factor that identifies the treatment groups is included in the model.
